# IGF2BP3 (IMP3) expression in angiosarcoma, epithelioid hemangioendothelioma, and benign vascular lesions

**DOI:** 10.1186/s13000-020-00951-x

**Published:** 2020-03-23

**Authors:** Misuzu Okabayshi, Tatsuki R. Kataoka, Marina Oji, Satoko Mibayashi, Kentaro Odani, Atsushi Otsuka, Hironori Haga

**Affiliations:** 1 Department of Diagnostic Pathology, Kyoto University Hospital, Sakyo-ku, Kyoto, 606-8507 Japan; 2 Translational Research Department for Skin and Brain Diseases, Department of Dermatology, Kyoto University Hospital, Kyoto, Japan

**Keywords:** Angiosarcoma, Epithelioid hemangioendothelioma, Immunohistochemistry, IGF2BP3

## Abstract

**Background:**

Insulin-like growth factor-2 messenger RNA-binding protein 3 (IGF2BP3 or IMP3) is an oncofetal protein that is expressed in various cancer types, and its expression is often associated with poor prognosis. IGF2BP3 expression has not been fully settled in vascular lesions.

**Methods:**

We evaluated the expression of IGF2BP3 in malignant (angiosarcoma and epithelioid hemangioendothelioma [EHE]) and benign (hemangioma, granulation tissue cappilaries, and pyogenic granuloma) vascular lesions using immunohistochemistry. IGF2BP3 expression was scored as negative (0% of endothelial/neoplastic cells), equivocal (1–25%), or positive (> 26%).

**Results:**

Eight of 30 (26.7%) cases of angiosarcoma and two of five (40%) cases of epithelioid hemangioendothelioma were positive for IGF2BP3. In contrast, hemangiomas (10 cases) and granulation tissue capillaries (12 cases) were all negative for IGF2BP3, and some cases of pyogenic granuloma (six of 14 cases) was scored as equivocal. In angiosarcoma, IGF2BP3 expression was independent of age, gender, location, morphological pattern, prognosis, presence of metastatic foci, and PD-L1 expression.

**Conclusions:**

IGF2BP3 is a useful marker to distinguish between malignant and benign vascular lesions.

## Introduction

IGF2BP3 is an oncofetal protein that is highly expressed in fetal tissue and gonads but is rarely found in other adult benign tissues [1]. IGF2BP3 is expressed in a variety of carcinomas and sarcomas [2–8]. Among sarcomas, leiomyosarcoma and chondrosarcoma express IGF2BP3, whereas their benign counterparts, leiomyoma and chondroma, are negative [3, 5]. One previous report showed that two of five cases (40%) of angiosarcoma were positive for IGF2BP3 in tissue microarray samples [8].

Angiosarcoma is a malignant tumor of vascular endothelial origin and is associated with poor prognosis [9]. The tumor cells are immunohistochemically positive for vascular markers such as CD31, CD34, ERG, and podoplanin [10]. Surgery is the most common approach to treat angiosarcoma, and achieving surgically negative margins improves prognosis [11, 12]. Therefore, accurate evaluation of surgical margins is essential for management of cutaneous angiosarcoma patients. However, it is sometimes difficult to evaluate the surgical margins because there is no immunohistochemical marker to distinguish malignant vasoformative cells from reactive capillary vessels.

The aim of this study was to determine whether insulin-like growth factor-2 mRNA binding protein 3 (IGF2BP3 or IMP3) could be such a marker. Here, we examined 30 cases of angiosarcoma to determine whether IGF2BP3 could be a useful diagnostic marker by comparing its expression in various types of vascular lesions.

## Materials and methods

### Pathological specimens

We selected all the examined cases which had been fixed in 4% neutral buffered formalin for 12–72 h at room temperature and embedded in paraffin for this study. All selected cases were diagnosed between 2010 and 2019. Under this condition, 30 cases of angiosarcoma and five cases of epithelioid hemangioendothelioma (EHE) were identified in the diagnostic database of Kyoto University Hospital. For comparison, benign vascular lesions (10 hemangiomas, 14 pyogenic granulomas, and 12 granulation tissues with proliferating capillaries) were selected from the most recent diagnostic sign-out cases under the same fixation condition. The clinical information is summarized in Table 1. All samples Patients signed the “Kyoto University Hospital Informed Consent Form for the Non-therapeutic Use of Histopathological Materials”, and signed forms were uploaded into each electronic health record.

**Table 1:** Details of patients

(A) Angiosarcomas							
**Case**	**Gender**	**Age**	**Site**	**Lesion**	**Morphology**	**PD-L1 staining**	**IGF2BP3 (IMP3) staining pattern**
1	F	25	Soft tissue	Metastasis	Well	Negative	Positive
2	M	61	Skin	Local recurrence	Spindle	Negative	Positive
3	F	65	Skin	Local recurrence	Epithelioid	Negative	Positive
4	M	70	Skin	Primary	Well	Positive	Positive
5	M	77	Skeletal muscle	Primary	Epithelioid	Positive	Positive
6	M	80	Skin	Local recurrence	Well	Negative	Positive
7	M	93	Skin	Local recurrence	Well	Negative	Positive
8	F	29	Soft tissue	Primary	Spindle	Negative	Positive
9	M	32	Heart	Primary	Spindle	Negative	Equivocal
10	M	42	Soft tissue	Metastasis	Spindle	Negative	Equivocal
11	F	59	Subcutis	Metastasis	Epithelioid	Positive	Equivocal
12	F	59	Skin	Primary	Spindle	Negative	Equivocal
13	F	63	Skin	Local recurrence	Spindle	Positive	Equivocal
14	F	64	Breast	Primary	Epithelioid	Negative	Equivocal
15	M	66	Skin	Local recurrence	Well	Negative	Equivocal
16	M	70	Skin	Primary	Well	Negative	Equivocal
17	M	70	Skin	Primary	Spindle	Negative	Equivocal
18	M	73	Subcutis	Primary	Well	Positive	Equivocal
19	M	75	Skin	Primary	Well	Positive	Equivocal
20	F	77	Soft tissue	Primary	Spindle	Negative	Equivocal
21	M	82	Skin	Primary	Well	Negative	Equivocal
22	M	83	Skin	Local recurrence	Well	Negative	Equivocal
23	F	84	Skin	Local recurrence	Epithelioid	Positive	Equivocal
24	F	87	Skin	Primary	Well	Negative	Equivocal
25	M	88	Skin	Primary	Well	Negative	Equivocal
26	F	65	Skin	Primary	Well	Negative	Negative
27	M	75	Kidney	Primary	Spindle	Negative	Negative
28	M	78	Skin	Local recurrence	Well	Negative	Negative
29	M	86	Skin	Primary	Well	Positive	Negative
30	F	87	Skin	Primary	Spindle	Negative	Negative
(B) Epithelioid hemangioendotheliomas							
**Case**	**Gender**	**Age**	**Site**	**IGF2BP3 (IMP3) staining pattern**			
1	M	23	Cerebrum	Positive			
2	M	81	Soft tissue	Positive			
3	M	27	Bone	Equivocal			
4	M	65	Esophagus	Equivocal			
5	F	33	Liver	Negative			
(C) Hemangiomas							
**Case**	**Gender**	**Age**	**Site**	**IGF2BP3 (IMP3) staining pattern**			
1	F	42	Lung	Negative			
2	F	45	Skin	Negative			
3	M	58	Skin	Negative			
4	F	64	Skin	Negative			
5	M	69	Skin	Negative			
6	F	69	Soft tissue	Negative			
7	F	80	Soft tissue	Negative			
8	M	80	Tongue	Negative			
9	M	82	Skin	Negative			
10	M	85	Skin	Negative			
(D) Pyogenic granuloma							
**Case**	**Gender**	**Age**	**Site**	**IGF2BP3 (IMP3) staining pattern**			
1	F	30	Lip	Equivocal			
2	F	63	Skin	Equivocal			
3	M	65	Nasal cavity	Equivocal			
4	M	65	Skin	Equivocal			
5	M	65	Skin	Equivocal			
6	M	78	Tongue	Equivocal			
7	M	33	Skin	Negative			
8	F	42	Soft tissue	Negative			
9	M	48	Skin	Negative			
10	M	66	Skin	Negative			
11	M	66	Skin	Negative			
12	M	67	Skin	Negative			
13	M	75	Tongue	Negative			
14	M	78	Skin	Negative			
(E) Granulation tissue							
**Case**	**Gender**	**Age**	**Site**	**IGF2BP3 (IMP3) staining pattern**			
1	F	34	Skin	Negative			
2	F	41	Skin	Negative			
3	F	46	Skin	Negative			
4	F	51	Skin	Negative			
5	M	57	Skin	Negative			
6	M	60	Skin	Negative			
7	F	68	Soft tissue	Negative			
8	M	74	Subcutis	Negative			
9	M	75	Buccal mucosa	Negative			
10	F	80	Skin	Negative			
11	F	82	Lip	Negative			
12	M	86	Mandible	Negative			

### Immunohistochemical analysis

Three-micrometer tissue sections were deparaffinized with xylene, rehydrated, and pretreated with 0.3% hydrogen peroxide for 5 min. For IGF2BP3 staining, after steam heating the sections for 20 min in pH 9.0 EDTA buffer, anti-IMP3 antibody (Ab; 1:75, mouse monoclonal clone 69.1, DAKO Cytomation, Glostrup, Denmark) was added and the sections were incubated for 15 min at room temperature following blocking of background staining using Protein Block (X0909; DAKO Cytomation). Staining was performed using a BOND III automated stainer (Leica Biosystems, Richmond, IL, USA) according to the manufacturer’s instructions. For PD-L1 staining, after steam heating the sections for 60 min in pH 8.5 citrate buffer, anti-PD-L1 Ab (1:200, rabbit monoclonal clone E1L3N, Cell Signaling Technology, Beverly, MA, USA) was added and the sections were incubated for 16 min at room temperature following blocking of background staining using Protein Block. Staining was performed using a Benchmark Ultra automated stainer (Ventana Medical Systems, AZ, USA) according to the manufacturer’s instructions. Stained sections were imaged under a BX45 microscope (Olympus, Tokyo, Japan) equipped with a DP26 digital camera (Olympus).

The degree of IGF2BP3 staining was scored, according to the proportion of the staining and regardless of the intensity of the staining as follows: negative (0% positive among endothelial/neoplastic cells), equivocal (1–25% positive), and positive (> 26%). PD-L1 staining was defined as positive if > 5% of membranous expression was observed at the tumor site, as reported previously [13].

### Statistical analysis

Differences between groups were examined for statistical significance using Student’s *t*-test or Chi-squared test (Microsoft Excel, Redmond, WA, USA). A *P* value less than 0.05 indicated statistical significance.

## Results

### Expression of IGF2BP3 in angiosarcoma

**Fig. 1:**
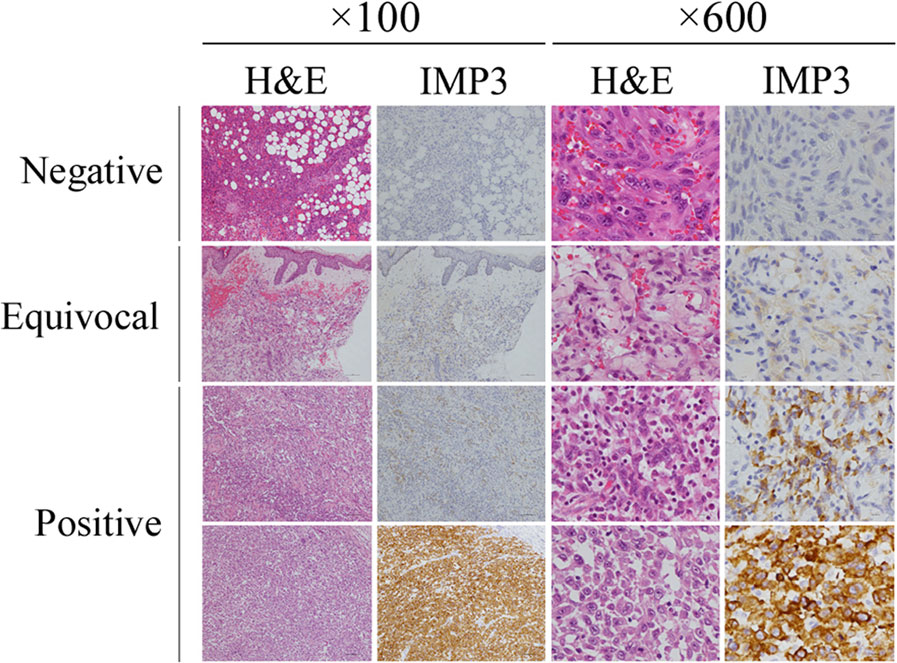
IGF2BP3 (IMP3) is expressed in some pathological samples of angiosarcoma; four representative cases are presented here. Immunohistochemistry (× 100 and × 600)

IGF2BP3 staining was positive in eight of 30 angiosarcoma cases (26.6%; Table 1a and Fig. 1). Most cases (*n* = 17, 56.6%) were scored as equivocal (Table 1a and Fig. 1). Completely negative staining was seen in five cases (16.6%; Table 1a and Fig. 1). There was no difference in clinical parameters (age, gender, location, morphological classification, presence of metastatic foci, and local recurrence) between IGF2BP3-positive and –equivocal /-negative cases (Table 2).

**Table 2:** Summary of clinical parameters of angiosarcomas

	IGF2BP3 (IMP3)-positive (*n* = 8)	IGF2BP3 (IMP3)-equivocal or –negative (*n* = 22)	Total (*n* = 30)	*P* value
**Age (y.o.)**	71.1 (25–93)	67.9 (32–88)	70.3 (25–93)	0.64*
**Gender (F:M)**	5:3	13:9	18:12	0.87**
**Location (Skin vs Non-skin)**	5:3	15:7	20:10	0.77**
**Morphology (Well vs Non-well)**	4:4	11:11	15:15	1.00**
**Metastatic foci (Present vs Absent)**	2:6	5:17	7:23	0.90**
**Local recurrence (Present vs Absent)**	4:4	8:14	12:18	0.50**
**PD-L1 staning (Positive vs Negative)**	2:6	6:16	8:18	0.83**

*: Student’s *t*-test

**: Chi-squared tests

Next, we assessed the association between IGF2BP3 expression and PD-L1 expression, which is a positive prognostic marker for angiosarcoma [13]. Among IGF2BP3-positive cases of angiosarcoma, two cases (25.0%) were PD-L1-negative and six (75.0%) were PD-L1-positive. Among negative or equivocal cases, one was a consultation from another hospital and no extra glass slide was available for PD-L1 analysis. Therefore, we assessed 21 angiosarcoma cases scored as negative or equivocal; four cases (19.0%) were PD-L1-negative and 17 (81.0%) were PD-L1-positive. Again, there was no statistical association between IGF2BP3 expression and PD-L1 expression among the angiosarcoma cases (Table 2).

### Expression of IGF2BP3 in epithelioid hemangioendothelioma (EHE)

**Fig. 2:**
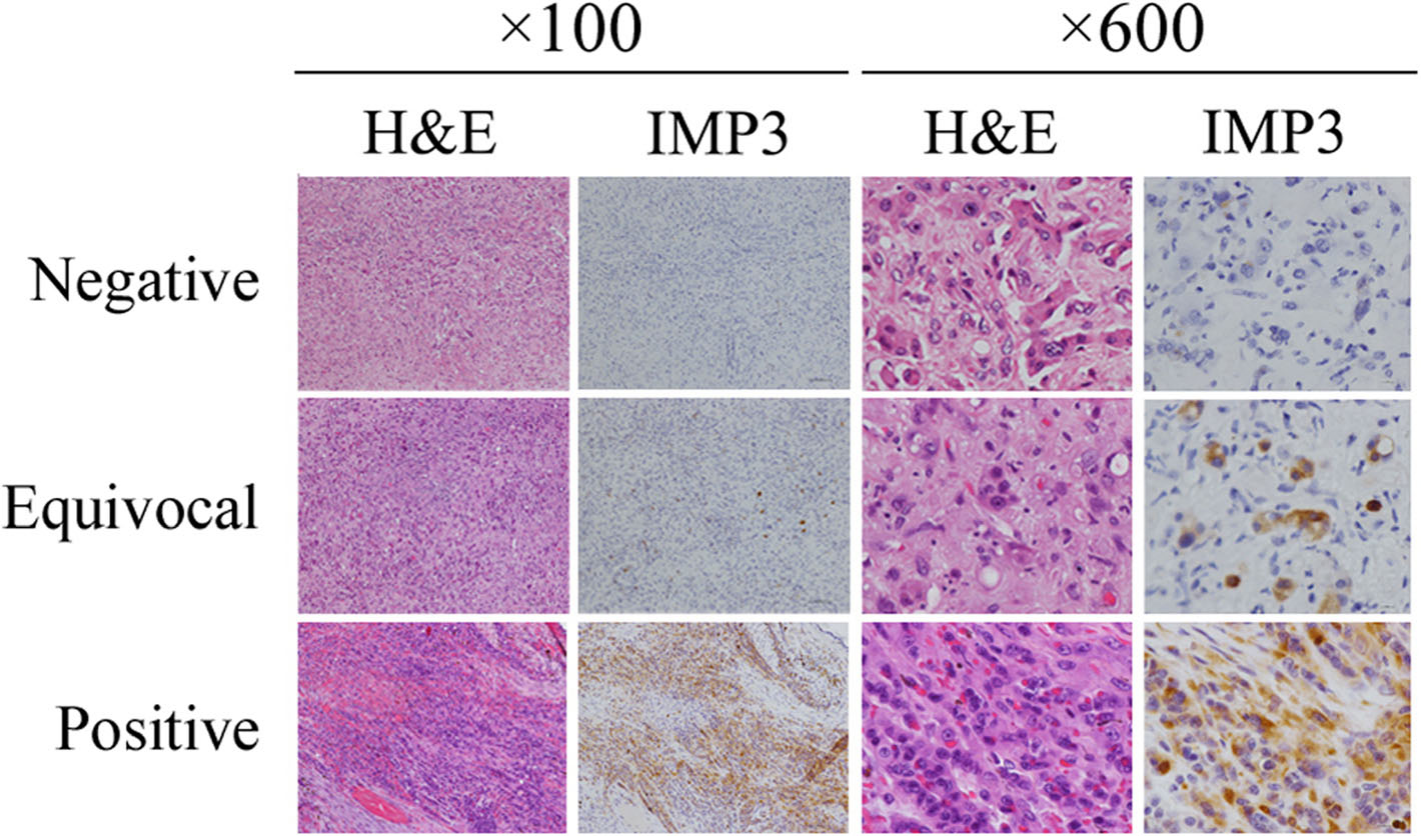
IGF2BP3 (IMP3) is expressed in some pathological samples of epithelioid hemangioendothelioma; three representative cases are presented here. Immunohistochemistry (× 100 and 600)

Two of five (40%) cases of EHE were scored as positive for IGF2BP3 (Table 1b and Fig. 2). The remaining cases were equivocal (*n* = 2) or negative (*n* = 1) (Table 1b and Fig. 2).

### Expression of IGF2BP3 in benign vascular lesions

**Fig. 3:**
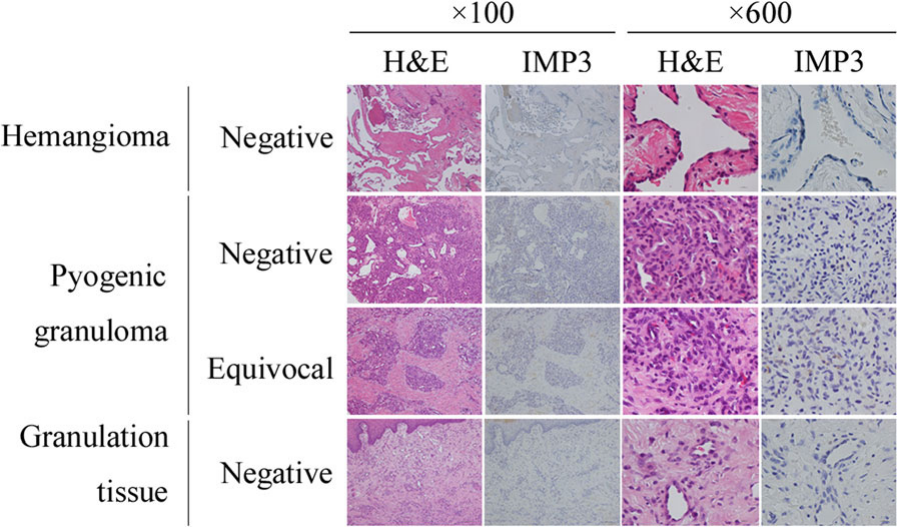
IGF2BP3 (IMP3) is negative or equivocally expressed in pathological samples of benign vascular lesions; four representative cases are presented here. Immunohistochemistry (× 100 and × 600)

No benign vascular lesion (*n* = 36) was scored as positive (Tables 1c – e and Fig. 3). Ten hemangioma cases and 14 granulation tissues showed completely negative staining (Tables 1c and e and Fig. 3). In contrast, six of 14 cases of pyogenic granuloma (42.9%) were scored as equivocal (Table 1d and Fig. 3).

## Discussion

IGF2BP3 (IMP3) is expected to be a marker to distinguish between malignant and benign lesions [2–8]. Here, we examined whether IGF2BP3 could distinguish between malignant and benign vascular lesions. We found eight of 30 angiosarcoma cases were positive for IGF2BP3, compatible with the previous study showing two of five cases of angiosarcoma were positive for IGF2BP3 in tissue microarray samples (26.7% vs 40.0%, *p* = 0.54) [8]. In addition, two of five (40%) cases of EHE were positive for IGF2BP3. In contrast to angiosarcoma and EHE, benign vascular lesions were all negative or equivocal for IGF2BP3. These results seem to show that IGF2BP3-positive vascular lesions are malignant, therefore the presence of IGF2BP3-positive vasoformative cells on the surgical margins of angiosarcoma and EHE should be judged as “margin positive”, and the followed additional resection. However, the utility of IGF2BP3 staining would be limited, because the majority of angiosarcoma and EHE cases are negative or equivocal for IGF2BP3.

The current study shows that benign vascular lesions, including hemangioma and granulation tissue, were negative for IGF2BP3. Surprisingly, some pyogenic granuloma samples were equivocal for IGF2BP3. Recently, pyogenic granuloma samples were reported to harbor BRAF and RAS mutations, suggesting that this may be a vascular tumor [14]. IGF2BP3-positive pyogenic granuloma might be associated with the presence of such mutations. IGF2BP3-equivocal stains were also observed in angiosarcoma (17 of 30 cases, 56.7%) and EHE (two of five cases, 40.0%). Further study will be necessary to determine the significance of the equivocal staining.

Here, we found that low proportions (< 3%) of non-neoplastic vascular cells, including benign vascular lesions, stained positive for IGF2BP3 (data not shown). IGF2BP3 is expressed in both malignant cells and non-neoplastic adult tissues such as germinal centers of lymphoid tissue [15]. In addition, IGF2BP3 expression is correlated with the aggressiveness or proliferative phenotypes of lymphoma [15–18]. IGF2BP3 is expressed in actively proliferating cells, whether neoplastic or non-neoplastic. In the current study, the proportion of IGF2BP3-positive cells was higher in neoplastic cells than in non-neoplastic lesions. To utilize IGF2BP3 staining as a marker for malignancy, the proportion of IGF2BP3 positivity in the examined lesions should be evaluated.

IGF2BP3 plays important roles in the RNA stabilization and translation of certain genes, including matrix metalloprotease (MMP)-9, high mobility group AT-hook 2 (HMGA2), and CD44 [19–21]. MMP9 and HMGA2 are associated with cell invasion and migration. IGF2BP3 promotes trophoblast invasion and migration via MMP9 mRNA stabilization and translation [19], and melanoma invasion and migration via HMGA2 mRNA stabilization and translation [20]. IGF2BP3 might promote angiosarcoma or EHE cell invasion and migration via the same mechanisms. CD44 is a cancer stem cell marker whose expression is correlated with pathogenesis in vascular tumors [21]. IGF2BP3 might also be correlated with the pathogenesis of vascular tumors via CD44 mRNA stabilization and translation.

## Conclusion

IGF2BP3 is a unique marker, indicating the vascular lesions as malignant when endothelial/neoplastic cells show positive by immunohistochemistry. We confirmed that most adult tissues were negative for IGF2BP3 and some actively proliferating vascular cells show equivocal expression. Further study will be necessary to determine the significance of the equivocal staining. Although sensitivity is not always high, IGF2BP3 can be a supplemental marker to recognize tumor cells in small biopsy specimens or tumor cut end for malignant vascular neoplasm.

## Data Availability

Is available upon request from the corresponding author.

## Abbreviations

Ab: Antibody
EHE: Epithelioid hemangioendothelioma
IGF2BP3: Insulin-like growth factor-2 messenger RNA-binding protein 3
HMGA2: High mobility group AT-hook 2
MMP: Matrix metalloprotease
